# The critical role of arginine residues in the binding of human monoclonal antibodies to cardiolipin

**DOI:** 10.1186/ar1449

**Published:** 2004-11-16

**Authors:** Ian Giles, Nancy Lambrianides, David Latchman, Pojen Chen, Reginald Chukwuocha, David Isenberg, Anisur Rahman

**Affiliations:** 1 Centre for Rheumatology, Department of Medicine, University College London, UK; 2 Medical Molecular Biology Unit, Institute of Child Health, University College London, UK; 3 Department of Medicine, Division of Rheumatology, University of California, Los Angeles, USA

**Keywords:** antiphospholipid antibodies, arginine, binding, cardiolipin

## Abstract

Previously we reported that the variable heavy chain region (V_H_) of a human beta_2 _glycoprotein I-dependent monoclonal antiphospholipid antibody (IS4) was dominant in conferring the ability to bind cardiolipin (CL). In contrast, the identity of the paired variable light chain region (V_L_) determined the strength of CL binding. In the present study, we examine the importance of specific arginine residues in IS4V_H _and paired V_L _in CL binding. The distribution of arginine residues in complementarity determining regions (CDRs) of V_H _and V_L _sequences was altered by site-directed mutagenesis or by CDR exchange. Ten different 2a2 germline gene-derived V_L _sequences were expressed with IS4V_H _and the V_H _of an anti-dsDNA antibody, B3. Six variants of IS4V_H_, containing different patterns of arginine residues in CDR3, were paired with B3V_L _and IS4V_L_. The ability of the 32 expressed heavy chain/light chain combinations to bind CL was determined by ELISA. Of four arginine residues in IS4V_H _CDR3 substituted to serines, two residues at positions 100 and 100 g had a major influence on the strength of CL binding while the two residues at positions 96 and 97 had no effect. In CDR exchange studies, V_L _containing B3V_L _CDR1 were associated with elevated CL binding, which was reduced significantly by substitution of a CDR1 arginine residue at position 27a with serine. In contrast, arginine residues in V_L _CDR2 or V_L _CDR3 did not enhance CL binding, and in one case may have contributed to inhibition of this binding. Subsets of arginine residues at specific locations in the CDRs of heavy chains and light chains of pathogenic antiphospholipid antibodies are important in determining their ability to bind CL.

## Introduction

The identification of antiphospholipid antibodies (aPL) is a key laboratory feature in the diagnosis of patients with antiphospholipid antibody syndrome (APS). The cardinal manifestations of this syndrome are vascular thrombosis, recurrent pregnancy loss, livedo reticularis and thrombocytopenia [1,2]. APS may affect any organ of the body, leading to a broad spectrum of manifestations [3]. It is the commonest cause of acquired hypercoagulability in the general population [4] and a major cause of pregnancy morbidity.

APS may occur as a 'freestanding' syndrome (primary APS) [5] or in association with other autoimmune rheumatic diseases (secondary APS) [6]. In both primary APS and secondary APS, recurrence rates of up to 29% for thrombosis and a mortality of up to 10% over a 10-year follow-up period have been reported [7]. The only treatment that reduces the risk of thrombosis in APS is long-term anticoagulation [8]. This treatment may have severe side effects, notably bleeding. It is therefore important to develop a greater understanding of how aPL interact with their target antigens so that new treatments for APS, which are both more effective and more accurately targeted to the causes of the disease process, may be developed.

aPL occur in 1.5–5% of healthy people and may also occur in various medical conditions without causing clinical features of APS [9]. The aPL that are found in patients with APS differ from those found in healthy people in that they target predominantly negatively charged phospholipid antibodies and are in fact directed against a variety of phospholipid binding serum proteins. These proteins include protein C, protein S, prothrombin and beta_2 _glycoprotein I (β_2_GPI) [10-13]. β_2_GPI is the most extensively studied of these proteins and appears to be the most relevant clinically [14-16]. Furthermore, high levels of IgG aPL, rather than IgM aPL, are closely related to the occurrence of thrombosis in APS [17,18].

Sequence analysis of human monoclonal aPL has shown that IgG aPL, but not IgM aPL, often contain large numbers of somatic mutations in their variable heavy chain region (V_H_) and variable light chain region (V_L_) sequences [19]. The distribution of these somatic mutations suggests that they have accumulated under an antigen-driven influence [20]. These monoclonal aPL tend to have accumulations of arginine residues, asparagine residues and lysine residues in their complementarity determining region (CDRs). Arginine residues have also been noted to play an important role in the CDRs of some murine monoclonal aPL [21,22].

Arginine residues, lysine residues and asparagine residues also occur very commonly in the CDRs of human and murine antibodies to dsDNA (anti-dsDNA) [23-25], particularly arginine residues in V_H _CDR3 [25-27]. It has been suggested that the structure of these amino acids allows them to form charge interactions and hydrogen bonds with the negatively charged DNA phosphodiester backbone [25,28]. We hypothesise that the same types of interaction may occur between negatively charged epitopes upon phospholipid antibodies/β_2_GPI and arginine residues, asparagine residues and lysine residues at the binding sites of high-affinity pathogenic IgG aPL.

We have previously described a system for the *in vitro *expression of whole IgG molecules from cloned V_H _and V_L _sequences of human monoclonal aPL antibodies [29]. This system was used to test the binding properties of combinations of heavy chains and light chains derived from a range of human antibodies. One of these antibodies, IS4, is an IgG antibody derived from a primary APS patient. IS4 binds to anionic phospholipid antibodies only in the presence of β_2_GPI, can bind to β_2_GPI alone and is pathogenic in a murine model [30]. It is therefore likely to be relevant in the pathogenesis of APS.

We found that the sequence of IS4V_H _was dominant in conferring the ability to bind cardiolipin (CL) while the identity of the V_L _paired with this heavy chain was important in determining the strength of CL binding [29].

Modelling studies have shown that multiple surface-exposed arginine residues were prominent features of the heavy chains and light chains that conferred the highest ability to bind CL. The CDR3 region of IS4V_H _contains five arginine residues, of which four are predicted by the model to be surface-exposed, and therefore is potentially important in binding to CL [29].

The purpose of the study reported in this paper was to define the contribution of different CDRs, and of individual arginine residues within those CDRs, in binding to CL. Patterns of CDR arginine residues in the cloned V_H _and V_L _sequences were altered by site-directed mutagenesis or by CDR exchange. The altered heavy chains and light chains were expressed transiently in COS-7 cells. Binding of the different heavy chain/light chain combinations to CL was tested by direct ELISA.

## Materials and methods

### Human monoclonal antibodies

IS4, B3 and UK4 are all human IgG monoclonal antibodies produced from lymphocytes of three different patients. IS4 was derived from a primary APS patient by the Epstein–Barr virus transformation of peripheral blood mononuclear cells and fusion with the human-mouse heterohybridoma K6H6/B5 cell line [31]. IS4 binds to CL in the presence of bovine and human β_2_GPI, and to human β_2_GPI alone [31]. B3 [32] and UK4 [33] were isolated by fusion of peripheral B lymphocytes from systemic lupus erythematosus patients with cells of the mouse human heteromyeloma line CB-F7. B3 binds single-stranded DNA, dsDNA, CL and histones [32,34]. UK4 binds negatively charged (but not neutral) phospholipid antibodies in the absence of β_2_GPI and does not bind DNA [33].

### Assembly of constructs for expression

#### Wild-type heavy chain and light chain constructs

Constructs containing the wild-type heavy chain and light chain were prepared as detailed fully in previous articles [29,35]. UK4V_H _could not be cloned into the appropriate plasmid, hence only UK4V_L _was available for analysis. The expression vectors (pLN10, pLN100 and pG1D210) were all kind gifts from Dr Katy Kettleborough and Dr Tarran Jones (Aeres Biomedical, London, UK).

#### Hybrid V_L _chain constructs

Each hybrid V_L _chain construct contained the CDR1 of one of the human monoclonal IgG antibodies IS4, B3 or UK4 and the CDR2 and CDR3 of a different one of these antibodies. Two hybrid V_L _chains (BU and UB) had previously been made by Dr Haley and colleagues [36], and a further four chains (IB, IU, BI and UI) were made by a similar method, as follows.

Two different wild-type V_L _expression vectors were digested with *Acc*65 I and *Pvu *I (Promega, Southampton, UK). *Acc*65 I cuts IS4, B3 or UK4 V_L _sequences at a position in FR2 that is 106 base pairs from the beginning of V_L_, but does not cut the expression vector outside the insert. *Pvu *I cuts the vectors at a single site approximately 1 kb downstream of the insert. Each vector was therefore digested into two linear bands; one of approximately 1.5 kb and the other of approximately 6 kb. The 1.5 kb fragment contained CDR2 and CDR3 of the IgG V_L _region and also part of the downstream expression vector containing the lambda constant region cDNA, while the 6 kb fragment contained CDR1 and the rest of the vector. The 6 kb fragment derived from one V_L _expression vector was ligated with the 1.5 kb fragment derived from the other. The resulting plasmid would contain CDR1 of one V_L _sequence and CDR2 and CRD3 of another V_L _sequence.

Since IS4, B3 and UK4 V_L _sequences differ in their content of the restriction sites *Aat *II and *Ava *I, we checked that the desired parts of each sequence were present in the new hybrid sequences by carrying out *Aat *II, *Hind *III/*Ava *I and *Aat *II/*Bam *HI digests.

#### Site-directed mutagenesis of IS4V_H_

We generated six mutant forms of IS4V_H _in which particular arginine residues were mutated to serine, using the QuikChange site-directed mutagenesis kit (Stratagene, La Jolla, CA, USA) according to the manufacturer's protocol. Serine was chosen because it is nonpolar. Germline reversion could not be performed because the exact germline D_H _gene of IS4V_H _CDR3 is unknown. Four mutants, named IS4V_H_i, IS4V_H_ii, IS4V_H_iii and IS4V_H_iv, contained single mutations of arginine residues at positions 96, 97, 100 and 100 g, respectively. The remaining two forms contained two arginine to serine mutations, at positions 96 and 97 in the IS4V_H_i&ii mutant and at all four sites in mutant IS4V_H_x.

### Expression of whole IgG molecules

The whole IgG molecules were expressed in COS-7 cells as described previously [29,37].

### Detection and quantitation of whole IgG molecules in COS-7 supernatant by ELISA

Whole IgG molecules were detected and quantitated in the COS-7 cell supernatants using a direct ELISA, as described in previous papers [29,35,37].

### Detection of binding to CL by ELISA

The binding of IgG molecules to CL was measured by direct ELISA as described previously [29].

## Results

### Sequences of light chains expressed

**Figure 1:**
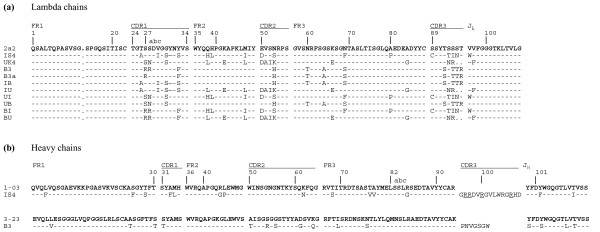
Sequence alignment of the expressed variable light chainregion (V_L_) and variable heavy chainregion (V_H_), using DNAplot software in VBASE. **(a) **Sequences of expressed V_λ _regions compared with gene 2a2. **(b) **Sequences of expressed V_H _regions compared with genes 1-03 (IS4) and 3–23 (B3). The D_H _regions could not be matched to germline genes. Arginine residues altered by site-directed mutagenesis to serine residues in IS4V_H _complementarity determining region (CDR) 3 are underlined. Amino acids are numbered according to Kabat. Dots inserted to facilitate the alignment. Dashes indicate homology with the corresponding germline sequence. FR, framework region.

Amino acid sequences of IS4V_L_, UK4V_L_, B3V_L _and germline gene 2a2 are shown in Fig. 1a. All of these light chains contain numerous somatic mutations. Previous statistical analysis has shown that the observed pattern of replacement mutations in the CDRs of these sequences is consistent with antigen-driven selection [32,33,35,38-40]. The light chain B3aV_L_, shown in Fig. 1a, was derived from B3V_L _by site-directed mutagenesis of Arg27a to serine [37].

The V_L _sequences of IS4, B3 and UK4 are all encoded by the germline V_λ _gene 2a2, but differ in their patterns of somatic mutation. B3V_λ _contains two adjacent arginine residues in CDR1, both produced by somatic mutations. UK4V_λ _has a single somatic mutation to arginine in CDR3 at position 94. A serine residue in CDR3 of IS4V_L _is replaced by asparagine.

Figure 1a also shows the amino acid sequences of the V_λ _CDR hybrids in which each newly formed chain construct contains CDR1 of one antibody with CDR2 and CDR3 of a different antibody. These hybrid sequences were named by combining the names of the two parent antibodies such that the first letter represented the antibody from which CDR1 was derived and the last letter represented the antibody from which both CDR2 and CDR3 were derived. Hybrid IB thus contains CDR1 from IS4, and CDR2 and CDR3 from B3, whereas hybrid BI contains the reverse combination (CDR1 from B3, and CDR2 and CDR3 from IS4).

### Sequences of heavy chains expressed

The amino acid sequences of IS4V_H _and B3V_H _chain and the corresponding germline genes are displayed in Fig. 1b. B3V_H _has a single somatic mutation to arginine in CDR2. The CDR2 of IS4V_H _contains an asparagine residue created by somatic mutation and in CDR3 there are multiple arginine residues, which are highly likely to have arisen as a result of antigen-driven influence. The four surface-exposed arginine residues that were mutated to serine to create the six mutant forms of IS4V_H _are underlined in Fig. 1b.

### Expression of whole IgG

Each of the 10 light chains shown in Fig. 1a was paired with B3V_H _and IS4V_H_. Each of the six mutant forms of IS4V_H _was paired with IS4V_L _and B3V_L_. A total of 32 heavy chain/light chain combinations were expressed in COS-7 cells. At least two expression experiments were carried out for each combination. IgG was obtained in the supernatant for all of the combinations.

The range of concentrations of IgG obtained in COS-7 cell supernatants, determined by ELISA, from each of the 32 heavy chain/light chain combinations are presented in Table 1. Identical concentrations were obtained for the combination IS4V_H_ii/B3V_L _from two different expression experiments. In each case the negative control sample, in which COS-7 cells were electroporated without any plasmid DNA, contained no detectable IgG. Consistently high yields were obtained with the B3V_H_/BIV_L_, B3V_H_/UIV_L _and IS4V_H_/UIV_L _combinations compared with the other antibody combinations. The phenomenon of variable expression with different V_H _and V_L _constructs is well documented both in this antibody expression system and in other systems [35,37], although the reason for the occurrence of variable expression is not clear.

### Results of anti-CL ELISA

**Figure 2:**
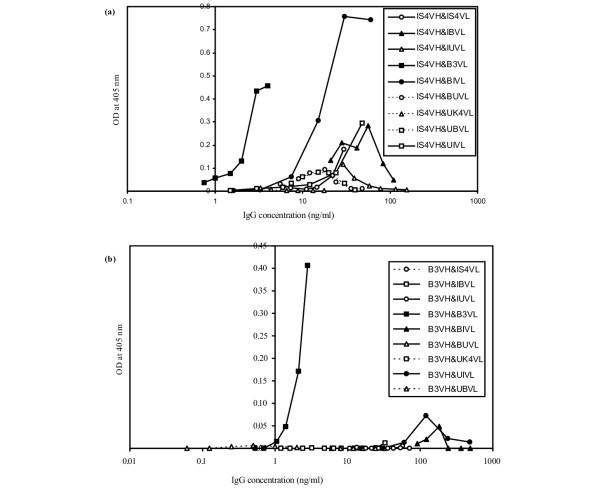
Effect of complementarity determining region exchange in the light chains. Cardiolipin binding of IgG in COS-7 cell supernatants containing wild-type heavy chains expressed with wild-type or hybrid light chain constructs. **(a) **Light chains expressed with IS4 variable heavy chainregion (V_H_). **(b) **Light chains expressed with B3V_H_. Presented as concentration of IgG in the supernatant versus optical density (OD) at 405 nm in the anti-cardiolipin ELISA.

**Figure 3:**
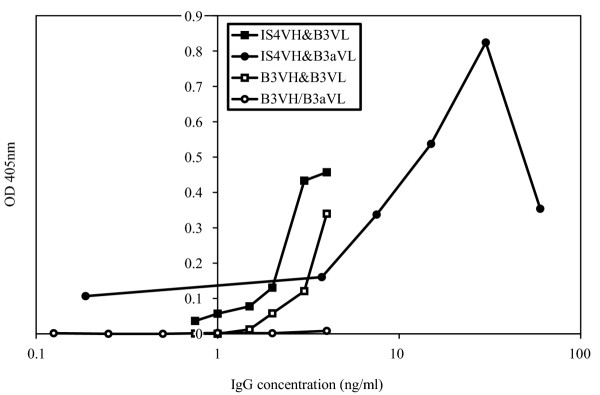
Effect of point mutation Arg27a to serine in B3 variable light chainregion (V_L_) complementarity determining region 1. Comparison of cardiolipin binding of IgG in COS-7 cell supernatants containing wild-type heavy chains expressed with B3V_L _or B3V_L_a. Presented as concentration of IgG in the supernatant versus optical density (OD) at 405 nm in the anti-cardiolipin ELISA.

**Figure 4:**
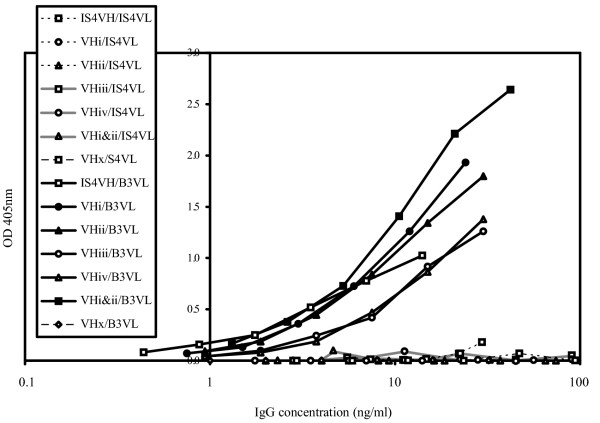
Effect of arginine to serine point mutations in IS4 variable heavy chainregion (V_H_) complementarity determining region 3. Cardiolipin binding of IgG in COS-7 cell supernatants containing wild-type or mutant forms of IS4 heavy chain expressed with wild-type B3 or IS4 light chains. The IS4V_H _mutants VHi, VHii, VHiii and VHiv contain single arginine to serine point mutations at positions 96, 97, 100 and 100 g, respectively; VHi&ii contains arginine to serine point mutations at positions 96 and 97; and VHx has an arginine to serine point mutation at all four positions. Presented as concentration of IgG in the supernatant versus optical density (OD) at 405 nm in the anti-cardiolipin ELISA.

For each heavy chain/light chain combination that bound CL, the linear portion of the binding curve for absorbance against antibody concentration was determined empirically, by dilution of antibody over a wide range of concentrations. Similar patterns of binding were obtained for each combination from repeated expression experiments, hence representative results from a single experiment only are shown in Figs 2,3,4.

#### The importance of arginine residues in IS4V_H_

As reported previously, the presence of the heavy chain of IS4 plays a dominant role in binding to CL [29]. IS4V_H _binds CL in combination with six of the 10 light chains tested (see Figs 2a and 3): B3V_L_, B3aV_L_, BIV_L_, IS4V_L_, IBV_L _and UIV_L_. Only one of these light chains (B3V_L_) binds CL in combination with B3V_H _(Fig. 2b).

To identify the features of IS4V_H _that enhance binding to CL, we focused on the combination IS4V_H_/B3V_L_. This combination shows high binding to CL. This binding could be altered by the replacement of some or all of the four surface-exposed arginine residues in IS4V_H _CDR3 to serine, as shown in Fig. 4. Substitution of all four arginine residues with serine residues (IS4V_H_x) abolished CL binding completely. This effect seems probably due entirely to the changes at positions 100 and 100 g. This is supported by the fact that heavy chain combinations containing arginine to serine mutations at these positions (IS4V_H_iii and IS4V_H_iv) displayed approximately 50% weaker binding to CL in combination with B3V_L _than did the wild-type IS4V_H_/B3V_L _combination. In contrast, there were no reductions in CL binding for the heavy chains containing arginine to serine mutations at position 96 (IS4V_H_i), at position 97 (IS4V_H_ii) or at both positions (IS4V_H_i&ii).

#### The importance of arginine residues in the light chain CDRs

Six light chains bound CL in conjunction with IS4V_H _(Figs 2a and 3). The strongest binding was seen with light chains containing B3V_L _CDR1, namely B3V_L_, B3aV_L _and BI V_L_, in combination with IS4V_H_. In contrast, light chains IB and UB, containing CDR2 and CDR3 from B3, showed weak binding and no binding to CL, respectively, in combination with IS4V_H_.

To test the hypothesis that the arginine at position 27a in B3V_L _CDR1 is responsible for the favourable effect of this CDR on binding to CL, we expressed combinations of IS4V_H _and B3V_H _with B3aV_L_, in which Arg27a has been mutated to serine. As shown in Fig. 3, there was a significant decrease in CL binding of B3V_H_/B3aV_L _compared with B3V_H_/B3V_L_. Although the combination IS4V_H_/B3aV_L _binds CL less strongly than does IS4V_H_/B3V_L_, reduction in binding is not as great as that seen when these light chains are combined with B3V_H_. This observation is consistent with the idea that IS4V_H _plays a dominant role in binding to CL.

Despite being tested at a range of concentrations up to 75 times higher than those that gave maximal CL binding for the other combinations containing IS4V_H_, none of the light chains containing CDR2 and CDR3 derived from UK4V_L_, including UK4 wild-type, IU and BU, showed any binding to CL.

## Discussion

Previously we have shown the important roles played in antigen binding by IS4V_H _and B3V_L_, which both contain multiple nongermline-encoded arginine residues in their CDRs, supporting the idea that this amino acid is important in creating a CL binding site [29]. The results described in the present study demonstrate that it is not just the presence of, but the precise location of arginine residues in the CDRs that is important in determining the ability to bind CL.

The importance of arginine residues at specific positions in the V_H _and V_L _sequences of anti-DNA antibodies has been examined by many groups, by expressing the antibodies *in vitro *and then altering the sequence of the expressed immunoglobulins by chain swapping or mutagenesis [27,37,41-43]. In general, these studies have shown that altering the numbers of arginine residues in the CDRs of these antibodies can lead to significant alterations in binding to DNA. Arginines in V_H _CDR3 often play a particularly important role in binding to this antigen [27,37,41-43]. Behrendt and colleagues recently demonstrated that the affinity of human phage-derived anti-dsDNA Fabs from a lupus patient correlated with the presence of somatically mutated arginine residues in CDR1 and CDR2 of the heavy chain [44].

Previous studies of the contribution of aPL heavy chains or light chains to CL binding have yielded conflicting results. Different groups have reported important contributions from the heavy chain [21,45], from the light chain [46], or from both chains [43,47]. In one of these studies the role of arginine residues was examined in a murine antibody (3H9) with dual specificity for phospholipid antibodies and DNA [21]. The introduction of arginine residues into the V_H _at positions known to mediate DNA binding enhanced binding to phosphatidylserine–β_2_GPI complexes and to apoptotic cell debris, which may be an important physiological source of both these antigens [48].

Our data show that combinations of IS4V_H _with light chains containing CDR1 of B3 (B3V_L_, B3aV_L _and BIV_L_) produced the strongest binding to CL. The CDR1 of B3V_L _and BIV_L _contains two surface-exposed arginine residues at positions 27 and 27a, while B3aV_L _contains only one arginine at position 27. Previous modelling studies have suggested that the binding of B3V_H_/B3V_L _to dsDNA is stabilised by the interaction of dsDNA with Arg27a in CDR1 and Arg54 in CDR2 of the light chain [34]. Expression and mutagenesis studies from our group confirmed that mutation of Arg27a to serine led to a reduction in binding to DNA [37]. In the present study the same change has been shown to reduce binding to CL, supporting the conclusion of Cocca and colleagues that arginines at particular positions can enhance binding to both DNA and CL [21].

It is important, however, not to overlook the possible contribution of other amino acids in B3V_L _to CL binding. For example, substitution of histidine at position 53 with lysine and substitution of serine at position 29 with glycine could significantly influence the stability of the antigen binding site. In fact, we have previously shown that introduction of the Ser29 to glycine mutation in addition to the Arg27a to serine mutation in the light chain of B3V_L_/B3V_H _leads to a further reduction in binding to dsDNA [37].

The presence of UK4V_L _CDR2 and CDR3 in any light chain blocked binding to CL, even when combined with B3V_L _CDR1 (light chain BU). UK4V_L_CDR1, however, does not block binding. We have previously shown that the presence of UK4V_L _CDR2 and CDR3 blocks binding to DNA and histones but not to the Ro antigen [36,37]. Modelling studies have shown that an arginine at position 94 in CDR3 of UK4V_L _hinders DNA binding sterically. A similar effect may be occurring with regards to the binding of UK4V_L _to CL.

The effect of point mutations of specific arginine residues in CDR3 of IS4V_H _upon CL binding is shown in Fig. 4. The low binding of IS4V_H_/IS4V_L _was abolished by inclusion of any one of these mutations. This is not the case, however, when these mutants are expressed with B3V_L_. In this case the arginine residues at 100 and 100 g confer a greater effect on CL binding compared with the arginine residues at positions 96 and 97. Substitutions of all four of these IS4V_H _CDR3 arginine residues were sufficient to completely abolish all binding to CL.

An accumulation of arginine residues in V_H _CDR3 has been noted in most, but not in all, sequences of pathogenic monoclonal aPL. From our detailed analysis of all published sequences of monoclonal aPL we found that of 13 monoclonal aPL that had been examined in various biological assays, eight monoclonal aPL had been shown to be pathogenic [49]. Three aPL derived from patients with primary APS and a healthy subject induced a significantly higher rate of foetal resorptions and a significant reduction in foetal and placental weight following intravenous injection into mated BALB/c mice [50,51]. Five other aPL derived from patients with primary APS and systemic lupus erythematosus/APS were found to be thrombogenic in an *in vivo *model of thrombosis [30]. We compared the sequences of these eight pathogenic antibodies with those of the other five antibodies, observing no evidence of pathogenicity in these bioassays. There was no evidence of preferential gene usage in either antibody group and somatic mutations were common in both groups. The presence of arginine residues in V_H _CDR3, however, did differ between pathogenic aPL and nonpathogenic aPL. Six of the eight pathogenic aPL, but only one of five nonpathogenic aPL, contain at least two arginine residues in V_H _CDR3 [49].

Our data confirm that the effect of arginine residues on binding to CL is highly dependent on the positions that they occupy in the sequence. The precise location of arginine residues has been shown to be important in the binding of both murine and human anti-dsDNA to DNA in numerous studies [25,26,37]. Interestingly, Krishnan and colleagues have demonstrated a strong correlation between specificity for dsDNA and the relative position of arginine residues in V_H _CDR3 [52,53]. They reported that the frequency of arginine expression among murine anti-dsDNA antibodies was highest at position 100, and they postulate that the importance of this residue in binding to dsDNA lies in its position at the centre of the V_H _CDR3 loop in the structure of the antigen combining site [52]. Assuming that this loop would be projected outward from the antigen combining site, an arginine residue at position 100 would be located at the apex of the V_H _CDR3 loop.

## Conclusion

We have demonstrated the relative importance of certain surface-exposed arginine residues at critical positions within the light chain CDR1 and heavy chain CDR3 of different human monoclonal antibodies in conferring the ability to bind CL in a direct ELISA. It is now important to test the effects of sequence changes involving these amino acids on pathogenic functions of these aPL, by expressing the altered antibodies in larger quantities from stably transfected cells, and then testing them in bioassays.

## Abbreviations

aPL = antiphospholipid antibodies; APS = antiphospholipid syndrome; β_2_GPI = beta_2 _glycoprotein I; CDR = complementarity determining region; CL = cardiolipin; dsDNA = double-stranded DNA; ELISA = enzyme-linked immunosorbent assay; Fab = antigen-binding fragment; V_H _= variable heavy chainregion; V_L _= variable light chainregion.

## Competing interests

The author(s) declare that they have no competing interests.

## Authors' contributions

IG produced four hybrid light chains, participated in the production of the mutant heavy chains, antibody expression and study design, and drafted the manuscript. NL participated in the production of the mutant heavy chains and antibody expression. PC and RC produced the human monoclonal aPL IS4. DL and DI participated in study design and coordination. AR conceived of the study, and participated in its design and coordination. All authors read and approved the final manuscript.

## Figures and Tables

**Table 1:** The range of IgG concentrations (ng/ml) produced by expression of the 32 heavy chain/light chain combinations

Heavy chain	Light chain contributing CDR1	Light chain contributing CDR2 and CDR3	Light chain name	IgG concentration (ng/ml)
IS4	IS4	IS4	IS4	24–368
IS4	IS4	B3	IB	22–140
IS4	IS4	UK4	IU	70–194
IS4	B3	B3	B3	5–14
IS4	B3	IS4	BI	50–60
IS4	B3	UK4	BU	5–60
IS4	UK4	UK4	UK4	11–22
IS4	UK4	IS4	UI	50–480
IS4	UK4	B3	UB	9–50
B3	IS4	IS4	IS4	71–192
B3	IS4	B3	IB	41–96
B3	IS4	UK4	IU	89–376
B3	B3	B3	B3	3.5–6
B3	B3	IS4	BI	120–608
B3	B3	UK4	BU	40–68
B3	UK4	UK4	UK4	8–28
B3	UK4	IS4	UI	60–480
B3	UK4	B3	UB	2–20
IS4	B3(Arg27aSer)	B3	B3a	48–60
B3	B3(Arg27aSer)	B3	B3a	2.5–4
IS4V_H_i	IS4	IS4	IS4	50–56
IS4V_H_ii	IS4	IS4	IS4	65–70
IS4V_H_iii	IS4	IS4	IS4	48–90
IS4V_H_iv	IS4	IS4	IS4	48–90
IS4V_H_x	IS4	IS4	IS4	78–94
IS4V_H_i&ii	IS4	IS4	IS4	74–80
IS4V_H_i	B3	B3	B3	24–54
IS4V_H_ii	B3	B3	B3	30
IS4V_H_iii	B3	B3	B3	30–34
IS4V_H_iv	B3	B3	B3	28–30
IS4V_H_x	B3	B3	B3	32–34
IS4V_H_i&ii	B3	B3	B3	32–47

IgG concentrations in COS-7 cell supernatants were determined by ELISA. The hybrid light chains were named by combining the names of the two parent antibodies such that the first letter represented the antibody from which the complementarity determining region (CDR) 1 was derived and the last letter represented the antibody from which both the CDR2 and CDR3 were derived. At least two expression experiments were carried out for each combination; identical concentrations were obtained for IS4V_H_ii/B3V_L _from two different expression experiments.
